# miR-10a/b-5p-NCOR2 Regulates Insulin-Resistant Diabetes in Female Mice

**DOI:** 10.3390/ijms251810147

**Published:** 2024-09-21

**Authors:** Se Eun Ha, Rajan Singh, Byungchang Jin, Gain Baek, Brian G. Jorgensen, Hannah Zogg, Sushmita Debnath, Hahn Sung Park, Hayeong Cho, Claudia Marie Watkins, Sumin Cho, Min-Seob Kim, Moon Young Lee, Tae Yang Yu, Jin Woo Jeong, Seungil Ro

**Affiliations:** 1 Department of Physiology and Cell Biology, University of Nevada School of Medicine, Reno, NV 89557, USA; seeunh@med.unr.edu (S.E.H.); jin.jbc@gmail.com (B.J.); gbaek@unr.edu (G.B.); hannahphillips@unr.edu (H.Z.); hansungp@unr.edu (H.S.P.); suminc@unr.edu (S.C.); 2 Department of Physiology, Wonkwang Digestive Disease Research Institute & Institute of Wonkwang Medical Science, School of Medicine, Wonkwang University, Iksan 54538, Republic of Korea; 1@wku.ac.kr (M.-S.K.); lmy6774@wku.ac.kr (M.Y.L.); 3 Division of Endocrinology and Metabolism, Department of Medicine, Wonkwang University School of Medicine, Iksan 54538, Republic of Korea; endocrinology1@wku.ac.kr (T.Y.Y.); jinu84@wkuh.org (J.W.J.); 4 RosVivo Therapeutics, Applied Research Facility, 1664 N. Virginia St., Reno, NV 89557, USA

**Keywords:** microRNAs, diabetes mellitus, estrogen, pancreatic β-cells

## Abstract

Gender and biological sex have distinct impacts on the pathogenesis of type 2 diabetes (T2D). Estrogen deficiency is known to predispose female mice to T2D. In our previous study, we found that a high-fat, high-sucrose diet (HFHSD) induces T2D in male mice through the miR-10b-5p/KLF11/KIT pathway, but not in females, highlighting hormonal disparities in T2D susceptibility. However, the underlying molecular mechanisms of this hormonal protection in females remain elusive. To address this knowledge gap, we utilized ovariectomized, estrogen-deficient female mice, fed them a HFHSD to induce T2D, and investigated the molecular mechanisms involved in estrogen-deficient diabetic female mice, relevant cell lines, and female T2D patients. Initially, female mice fed a HFHSD exhibited a delayed onset of T2D, but ovariectomy-induced estrogen deficiency promptly precipitated T2D without delay. Intriguingly, insulin (INS) was upregulated, while insulin receptor (INSR) and protein kinase B (AKT) were downregulated in these estrogen-deficient diabetic female mice, indicating insulin-resistant T2D. These dysregulations of INS, INSR, and AKT were mediated by a miR-10a/b-5p-NCOR2 axis. Treatment with miR-10a/b-5p effectively alleviated hyperglycemia in estrogen-deficient T2D female mice, while β-estradiol temporarily reduced hyperglycemia. Consistent with the murine findings, plasma samples from female T2D patients exhibited significant reductions in miR-10a/b-5p, estrogen, and INSR, but increased insulin levels. Our findings suggest that estrogen protects against insulin-resistant T2D in females through miR-10a/b-5p/NCOR2 pathway, indicating the potential therapeutic benefits of miR-10a/b-5p restoration in female T2D management.

## 1. Introduction

Type 2 diabetes (T2D) is caused by a combination of insulin resistance and an inability of pancreatic β-cells to produce sufficient insulin [1]. T2D develops twice as often in males compared to females [2]. Sex hormones exert differential effects on female and male diabetic patients. The observed lower prevalence of T2D in females [3] suggests that female sex hormones could potentially be protective against β-cell injury. Increasing evidence from both human and animal studies shows that estrogen has antidiabetic properties [3,4]. Premenopausal female mice are protected from β-cell apoptosis and hyperglycemia, but male mice develop insulin-deficient diabetes [3,5,6,7]. Additionally, β-estradiol (E2), the main estrogen hormone produced by the ovaries, prevents proinflammatory cytokine-induced apoptosis in isolated human pancreatic islets [8]. Estrogen mediates profound effects throughout the body, manifesting antidiabetic actions in both humans and rodents [9,10,11]. The actions of estrogen are facilitated via two nuclear estrogen receptors, ERα and ERβ, which function as ligand-activated transcription factors [12]. Estrogens in female mice protect against the development of T2D by promoting the degradation of misfolded insulin protein, which does not occur in male mice [13]. In human patients, post-menopausal hormone therapy utilizing estrogens reduced the incidence of T2D in post-menopausal females [14]. These observations and clinical findings suggest that estrogens play an important role in regulating glucose homeostasis.

microRNAs (miRNAs), repressing gene expression by binding to sequence-targeted mRNAs, serve as master regulators of cell differentiation, proliferation, and apoptosis [15]. In our previous study, we discovered that both miR-10a-5p and miR-10b-5p (miR-10a/b-5p) regulate the onset of T2D through modulation of the receptor tyrosine kinase (KIT) in murine pancreatic β-cells [16]. Our findings underscored the essential role of miR-10a/b-5p in the growth and functionality of pancreatic KIT^+^ β-cells in mice [16]. We demonstrated that the deficiency of miR-10b-5p in KIT^+^ β-cells triggers T2D onset in male mice, but not in females, suggesting a potential protective effect of estrogen against T2D onset in females lacking miR-10b [16]. However, the molecular mechanism underlying the estrogen-protected T2D in females by miR-10a/b-5p is not fully explored.

In this study, we investigated the estrogen effects on the onset of T2D in ovariectomized (OVX) female mice, compared to male mice, both subjected to a high-fat, high-sucrose diet (HFHSD). Furthermore, we explored the potential therapeutic effects of the antidiabetic miR-10a/b-5p in rescuing T2D progression in OVX/HFHSD-fed female mice. Through comprehensive analysis, we characterized key biomarkers associated with insulin resistance and glucose homeostasis within the estrogen and miR-10a/b-5p pathway in OVX/T2D female mice, extending our findings to include human female patient samples with T2D. Our results demonstrate that miR-10a/b-5p modulates insulin-resistant T2D through an estrogen-dependent mechanism in females.

## 2. Results

### 2.1. HFHSD-Fed Female Mice Delay the Development of T2D

**Figure 1:**
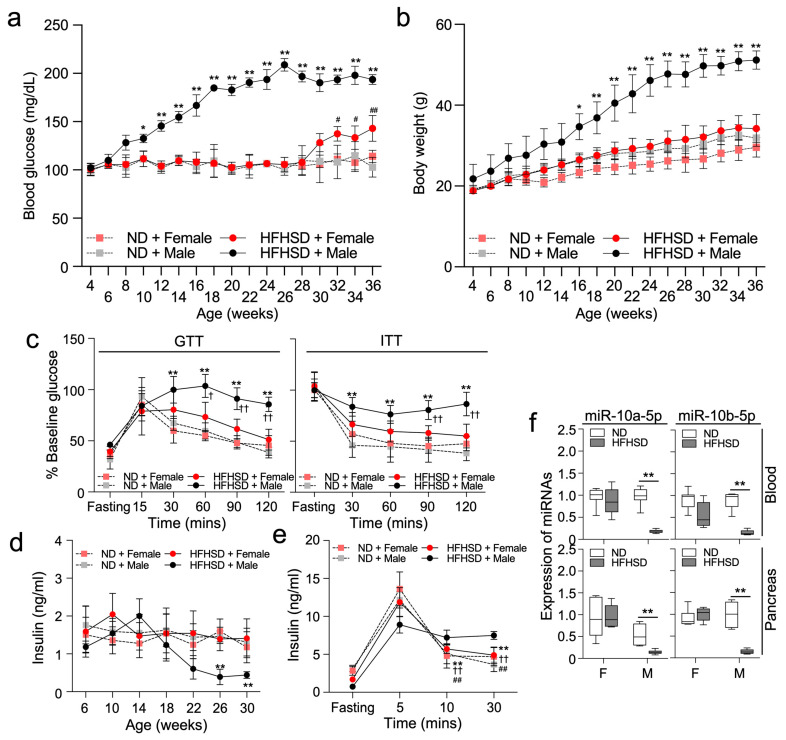
Male mice, but not female mice exhibit a diabetic phenotype when fed a high-fat high-sucrose diet (HFHSD). Male and female C57 mice were fed either a normal diet (ND) or a HFHSD. (**a**,**b**) Comparison of fasting blood glucose levels and body mass. n = 7 per group. * *p* < 0.05 and ** *p* < 0.01 (ND versus HFHSD in male mice); *^#^ p* < 0.05 and *^##^ p* < 0.01 (ND versus HFHSD in female mice). (**c**) Comparison of intraperitoneal glucose tolerance tests (GTT) and insulin tolerance tests (ITT) at 28 weeks old. n = 7 per group. ** *p* < 0.01 (ND versus HFHSD in male mice); ^†^ *p* < 0.05 and ^††^ *p* < 0.01 (versus HFHSD-fed female mice). (**d**) Changes in insulin levels after 6 h of fasting. n = 12 per group. ** *p* < 0.01 (ND versus HFHSD in male mice). (**e**) Comparison of insulin levels after 6 h of fasting and after glucose stimulation at 5 min, 10 min, and 30 min at 32 weeks old. n = 5 per group. ** *p* < 0.01 (10 min, 30 min versus 5 min in fed HFHSD female mice); ^††^ *p* < 0.01 (10 min, 30 min versus 5 min in ND-fed female mice); *^##^ p* < 0.01 (10, 30 min versus 5 min in ND-fed male mice). (**f**) Expression profiles of miR-10a-5p and miR-10b-5p in the whole blood, and pancreas tissue of males and females fed a ND or a HFHSD for 28 weeks. n = 3–6 per group. ** *p* < 0.01 (ND versus HFHSD in male mice). For all panels, error bars indicate the standard error of the mean (SEM) derived from one-way ANOVA.

HFHSD-fed C57 male mice became hyperglycemic and gained significantly more body mass when compared to both male and female ND-fed mice (Figure 1a,b). However, HFHSD-fed female mice did not have significant increases in blood glucose levels or body mass until 28 weeks, followed by a gradual increase in glucose levels thereafter. Glucose tolerance and insulin tolerance tests (GTT and ITT) confirmed that HFHSD-fed male mice had typical symptoms of T2D: hyperglycemia and impaired insulin sensitivity (Figure 1c,d). In contrast, HFHSD-fed female mice had normal glucose levels at the same time point as ND-fed male mice (Figure 1c). Fasting insulin levels in HFHSD-fed male mice temporarily increased at 14 weeks and markedly decreased thereafter, while insulin levels did not significantly change in HFHSD-fed female mice up to 30 weeks (Figure 1d). Insulin levels after glucose stimulation in HFHSD-fed male mice increased and sustained the increased level longer than both ND-fed males and females, suggesting elevated insulin resistance in HFHSD-fed males (Figure 1e). Previously, we showed a significant decrease in the levels of miR-10a/b-5p in both HFHSD-fed T2D male mice and human patients with T2D [16]. Similarly, the levels of miR-10a-5p and miR-10b-5p in the whole blood and pancreas were markedly decreased in HFHSD-fed male mice, but not HFHSD-fed female mice (Figure 1f).

### 2.2. Ovariectomized HFHSD-Fed Female Mice Develop T2D without Delay

**Figure 2:**
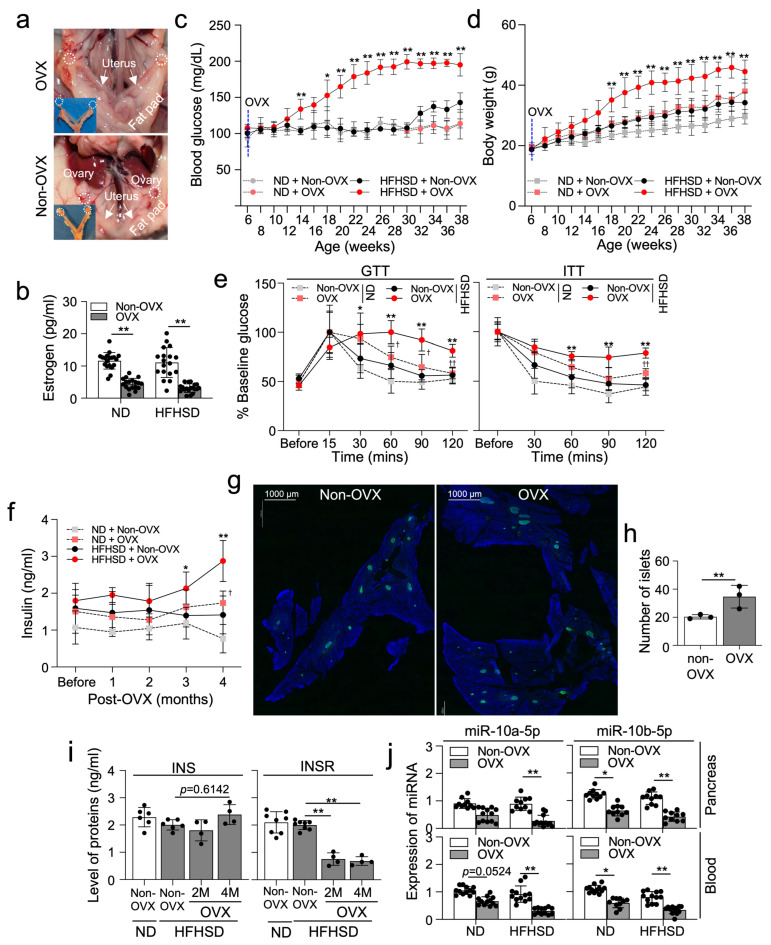
Ovariectomized (OVX) female mice fed a HFHSD develop diabetes and obesity. (**a**) Gross anatomical images of OVX or non-OVX C57 females. (**b**) Levels of estrogen (pg/mL) in the serum from OVX or non-OVX females fed a HFHSD or a ND. n = 12 per group. ** *p* < 0.01 (OVX versus non-OVX). (**c**,**d**) Comparison of fasting blood glucose levels and body mass in non-OVX or OVX female mice. n = 7 per group. * *p* < 0.05 and ** *p* < 0.01, OVX versus non-OVX. (**e**) Comparison of intraperitoneal GTT and ITT. n = 7 per group. * *p* < 0.05 and ** *p* < 0.01 (OVX versus non-OVX in HFHSD-fed female mice); ^†^ *p* < 0.05 and ^††^ *p* < 0.01 (ND versus HFHSD in OVX female mice). (**f**) Changes in insulin levels after 6 h of fasting in the serum in non-OVX and OVX females. * *p* < 0.05 and ** *p* < 0.01 (OVX versus non-OVX in HFHSD-fed female mice); ^†^ *p* < 0.05 (ND versus HFHSD in OVX female mice). n = 5 per group. (**g**) Cross-section images of the pancreatic islets containing β cells (insulin, INS^+^) from OVX and non-OVX HFHSD-fed female mice. Scale bars are 1000 μm. (**h**) Quantification of the number of islets in (**g**). n = 3 per group. ** *p* < 0.01 (OVX versus non-OVX). (**i**) Comparison of insulin (INS) and insulin receptor (INSR) levels in the pancreas tissue from OVX or non-OVX females fed a HFHSD or a ND. n = 4 per group. ** *p* < 0.01 (versus Non-OVX in HFHSD-fed female mice). (**j**) Expression profiles of miR-10a-5p and miR-10b-5p in the whole blood and pancreas of non-OVX or OVX female mice under ND or HFHSD conditions. * *p* < 0.05 and ** *p* < 0.01. n = 12 per group. Error bars indicate the SEM derived from one-way ANOVA.

C57 female mice were ovariectomized (OVX) (Figure 2a) and fed a ND or a HFHSD for 8 months, inducing T2D. Substantial reduction in total estrogen levels was confirmed in OVX mice fed either a HFHSD or a ND (Figure 2b). Blood glucose levels in OVX/HFHSD-fed female mice were notably increased earlier, observed at 14 weeks, compared to HFHSD-fed non-OVX mice, where blood glucose levels began to rise at 32 weeks (Figure 2c), indicating that estrogen protects the onset of T2D between 14–30 weeks in females under HFHSD conditions. Conversely, ND-fed OVX female mice showed no significant change in blood glucose levels, indicating that estrogen deficiency alone does not elevate blood glucose. Similarly, OVX/HFHSD-fed female mice showed a notable increase in body mass earlier than HFHSD-fed non-OVX mice (Figure 2d). However, both HFHSD and OVX in females led to a slight increase in body mass, suggesting that, in addition to HFHSD, estrogen deficiency induces an increase in body mass under ND conditions. The glucose tolerance test confirmed severe diabetes onset in OVX/HFHSD-fed mice at 22 weeks (4 months post-HFHSD) (Figure 2e). Additionally, the insulin tolerance test showed marked insulin resistance developed in these mice 4 months post-HFHSD (Figure 2e). We further confirmed abnormal increases in post-fasting insulin levels in both blood and pancreas of HFHSD-fed OVX mice 4 months post-HFHSD (Figure 2f). The number of pancreatic islets containing INS^+^ β-cells increased in OVX/HFHSD-fed mice (Figure 2g,h). Notably, the levels of insulin (INS) also increased in OVX/HFHSD-fed mice at 4 months, while the expression of insulin receptor (INSR) significantly decreased at 2 and 4 months (Figure 2i). These results indicate that the diabetic phenotype in OVX/HFHSD-fed mice is characterized by increased insulin resistance due to a reduction in INSR. Interestingly, the expression of miR-10a/b-5p decreased in both the blood and pancreas of OVX/HFHSD-fed mice, as well as in ND-fed OVX mice (Figure 2j), suggesting estrogen deficiency drives the reduction in these miRNA expressions in female mice regardless of dietary intervention.

### 2.3. β-Estradiol Partially Enhances Glucose and Insulin Homeostasis in Ovariectomized HFHSD-Fed Female Mice

**Figure 3:**
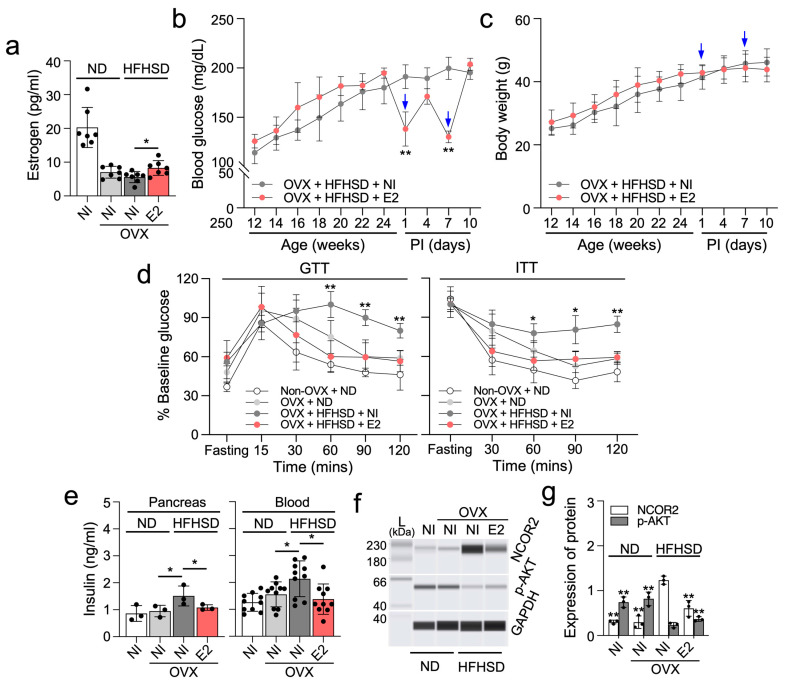
Estrogen partially improves glucose and insulin homeostasis in ovariectomized HFHSD-fed diabetic female mice. (**a**) Comparison of estrogen levels in OVX mice fed a ND or a HFHSD and injected twice with 50 µg/kg of β-estradiol (E2) at post-injection 1 week or given no injection (NI). n = 7 per group. (**b**,**c**) Fasting blood glucose levels and body mass in OVX mice fed a HFHSD and injected twice (↓) with E2 or given NI. n = 7 per group. * *p* < 0.05 and ** *p* < 0.01 (E2 versus NI). n = 5–10 per group. (**d**) Comparison of GTT and ITT after the 2^nd^ E2 injection. n = 7 per group. * *p* < 0.05 and ** *p* < 0.01 (E2 versus NI). (**e**) Comparison of insulin levels after 6 h of fasting in OVX mice fed a ND or HFHSD after the 2nd E2 injection. n = 3 (pancreas) or 7 (blood) per group. * *p* < 0.05. (**f**,**g**) Quantification of NCOR2 and phosphorylated-AKT (Ser473) in the pancreas of OVX female mice fed a ND or a HFHSD after the 2nd E2 injection. n = 3 per group. ** *p* < 0.01 (versus OVX mice fed HFHSD and given NI). Error bars indicate the SEM derived from one-way ANOVA.

We next investigated the impact of the primary estrogen hormone, β-estradiol (E2) on diabetes in OVX/HFHSD-fed female mice. Estrogen levels were reduced in OVX/HFHSD-fed female mice but partially restored with a 50 µg/kg E2 injection (Figure 3a). Following a single E2 injection, glucose levels exhibited a temporary decrease in OVX/HFHSD-fed female mice at 1 week post-injection, followed by a rebounding increase at 2 weeks post-injection (Figure 3b). However, OVX/HFHSD-fed female mice injected with E2 did not show a significant change in body mass and food intake (Figure 3c, Supplementary Table S1A,B). Notably, glucose and insulin tolerance improved in the E2–injected OVX/HFHSD-fed female mice (Figure 3d). Fasting insulin levels, abnormally increased in the pancreas tissue and blood of OVX/HFHSD-fed female mice, were reduced following E2 injection (Figure 3e). Insulin sensitivity is intricately regulated by several factors, including nuclear receptor corepressor 2 (NCOR2) and protein kinase B (AKT) signaling. NCOR2 has been identified as a negative regulator of insulin sensitivity [17,18], while AKT signaling exerts a positive influence [19]. Notably, NCOR2 is a direct target of miR-10a/b-5p [20]. The levels of NCOR2 in the pancreas of OVX/HFHSD-fed female mice significantly increased, but decreased after E2 injection while phosphorylated (activated) AKT (p-AKT, Ser473) levels showed an inverse correlation with NCOR2 expression (Figure 3f,g). This result suggests that insulin sensitivity is impaired in OVX/HFHSD-fed female mice but improved by E2 through modulation of the NCOR2-AKT axis.

### 2.4. miR-10a/b-5p Effectively Alleviate Diabetes in OVX/HFHSD-Fed Female Mice

**Figure 4:**
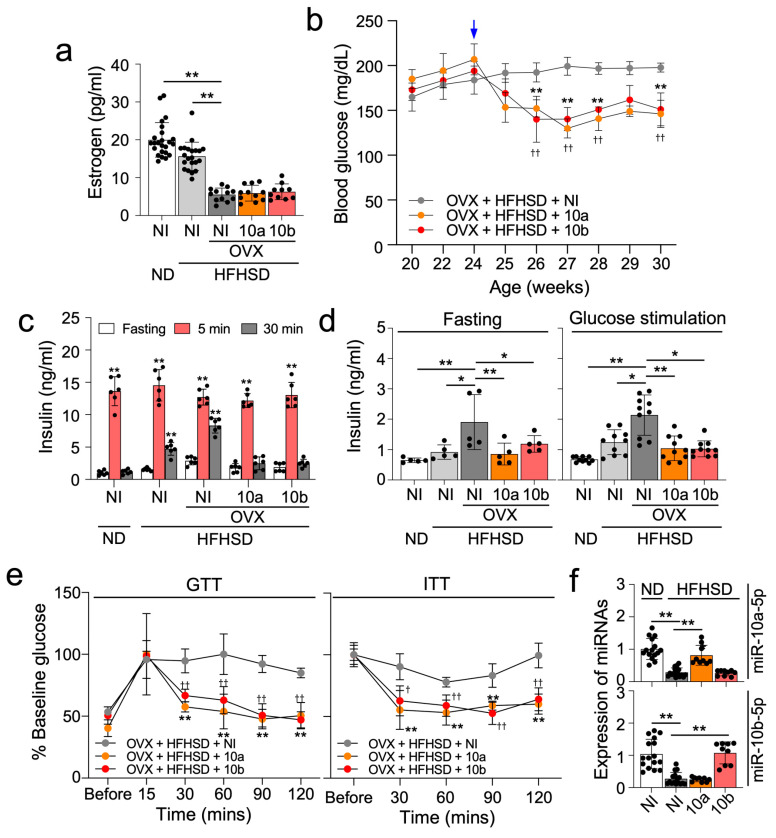
miR-10a-5p mimic and miR-10b-5p mimic rescue diabetic phenotype in ovariectomized HFHSD-fed diabetic female mice. (**a**) Levels of estrogen (pg/mL) in the serum from OVX mice fed a ND or a HFHSD and injected once with 500 ng/g of miR-10a-5p mimic (10a), miR-10b-5p mimic (10b) or given no injection (NI). n = 20 per group. (**b**) Fasting blood glucose levels in OVX mice fed a ND or a HFHSD and injected (↓) with 10a, 10b, or given NI. n = 7 per group. ** *p* < 0.01 (10a versus NI); ^††^ *p* < 0.01 (10b versus NI). n = 7–10 per group. (**c**) Insulin levels after glucose administration at 5 min, 10 min, and 30 min after 6 h of fasting in OVX and ND or HFHSD-fed females at 3 weeks post-injection (PI) in (**b**). n = 6 per group. ** *p* < 0.01 (5 min, 30 min versus 5 min). (**d**) Comparison of insulin levels after 6 h of fasting and 2 h after glucose stimulation in OVX and ND or HFHSD-fed females at 3 weeks PI. n = 5 per group. * *p* < 0.05 and ** *p* < 0.01 (versus OVX female given NI in HFHSD). (**e**) Comparison of GTT and ITT at 3 weeks PI. n = 7 per group. ^†^ *p* < 0.05, ^††^ *p* < 0.01 and ** *p* < 0.01 (versus OVX female given NI in HFHSD). (**f**) Levels of miR-10a-5p and miR-10b-5p in pancreas at 3 weeks PI. n = 10 per group. ** *p* < 0.01 (versus OVX female NI in HFHSD). For all panels, error bars indicate the SEM derived from one-way ANOVA.

We previously showed that the reintroduction of miR-10a-5p or miR-10b-5p rescued the diabetic phenotype in HFHSD-induced T2D male mice [16]. In this study, we similarly tested the effects on T2D of miR-10a/b-5p in OVX/HFHSD-fed female mice. Notably, OVX/HFHSD-fed female mice exhibited decreased estrogen levels, but an injection of miR-10a/b-5p did not influence estrogen levels (Figure 4a). A single injection of miR-10a/b-5p in OVX/HFHSD-fed female mice substantially lowered glucose levels for up to 6 weeks (Figure 4b). However, OVX/HFHSD-fed female mice injected with miR-10a/b-5p did not show a significant change in body weight and food intake (Supplementary Table S1A,B). Glucose-stimulated insulin levels in ND-fed female mice showed an insulin spike at 5 min followed by a marked decrease to the basal level at 30 min (Figure 4c). However, the insulin levels were sustained longer in OVX/HFHSD-fed female mice than in non-OVX/ND-fed female mice and non-OVX/HFHSD-fed female mice (Figure 4c), suggesting that a combination of estrogen deficiency and HFHSD could lead to insulin resistance. Insulin spiking and usage were improved by miR-10a/b-5p injection in OVX/HFHSD-fed female mice, indicating that miR-10a/b-5p could reduce insulin resistance (Figure 4c). Insulin levels 2 h post-glucose stimulation were significantly increased in OVX/HFHSD-fed female mice but decreased by miR-10a/b-5p injection in OVX/HFHSD-fed female mice (Figure 4d). Glucose and insulin tolerance in OVX/HFHSD-fed female mice were also significantly improved by miR-10a/b-5p injection (Figure 4e). miR-10a/b-5p levels were markedly decreased in OVX/HFHSD-fed female mice, but substantially restored by miR-10a/b-5p injection, respectively (Figure 4f).

### 2.5. β-Estradiol (E2) Positively Regulates INSR via a Regulatory Loop of NCOR2-miR-10a-5p

**Figure 5:**
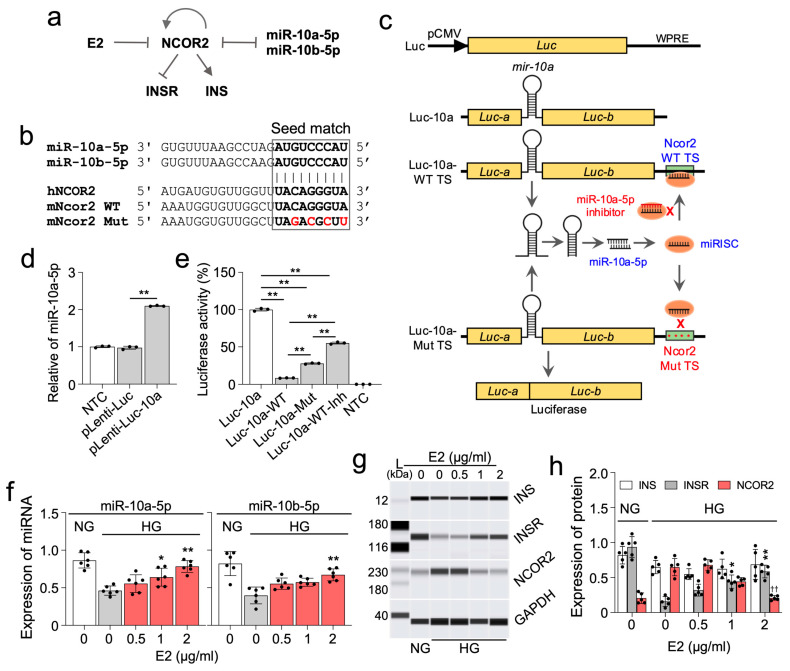
β-estradiol (E2) regulates the expression of essential metabolic proteins via miR-10a/b-5p. (**a**) Ingenuity Pathway Analysis of the target genes of miR-10a/b-5p and E2. (**b**) Seed match target sequences of miR-10a/b-5p in the 3′ UTR of NCOR2 in humans and mice. (**c**) Schematic illustration of the target validation mechanism and maps of the pLenti-Luc-10a-Ncor2 vectors. In this design, the mouse pre-mir-10a (110 bp) with an artificial intron is strategically inserted within the luciferase (*Luc*) gene, resulting in separated Luc-a and Luc-b exons. The wild type (WT) or mutant (Mut) target-binding sequence of the mouse Ncor2 is then introduced into the 3′ UTR of the *Luc* gene. Upon transcription, a miR-10a duplex comprising mature miR-10a-5p and miR-10a-3p strands is generated from the artificial intron, which encodes a pre-mir-10a spliced out from the primary Luc-a and Luc-b transcripts. The miR-10a-5p strand is preferentially selected within the RNA-induced silencing complex (RISC), forming the miRISC that specifically binds to the Ncor2 WT target site (TS), but not the Mut TS. The exonic segments of Luc-a and Luc-b then join to produce functional LUC protein (luciferase), whose activity is downregulated by the targeting action of miR-10a-5p. Moreover, inhibition of miR-10a-5p by its inhibitor mitigates its targeting effect on luciferase. *Luc*, luciferase gene; pCMV, CMV promoter; WPRE, Woodchuck Hepatitis Virus Posttranscriptional Regulatory Element DNA sequence; miRISC, miRNA-RNA-induced silencing complex. (**d**) Expression of miR-10a-5p in HEK293T cells transfected with the luciferase reporter plasmid containing the murine pre-mir-10a insertion (pLenti-Luc-10a) and without insertion (pLenti-Luc). pLenti-Luc-10a vector was used to insert the NCOR2 wild type target site (WT TS) or the mutant target site (Mut TS) at the end of luciferase gene (c). n = 3 per group. (**e**) Luciferase activity in HEK293T cells transfected with pLenti-Luc-10a, pLenti-Luc-10a-WT TS, pLenti-Luc-10a-Mut TS, or co-transfected with miR-10b-5p inhibitor (pLenti-Luc-10a-WT TS-Inh). NTC, non-transfected cells. n = 3 per group. (**f**) Effects of E2 on expression of miR-10a/b-5p in NIT-2 cells. n = 5–6 per group. (**g**,**h**) Automated western blots and quantified protein expression of INS, INSR, and NCOR2 in NIT-2 cells cultured in a normal glucose medium (1 mg/L, NG) or a high glucose medium (10 mg/L, HG) and treated with E2 for 24 h. A protein marker (L) with corresponding molecular weights (kDa) is shown. n = 5 per group. * *p* < 0.05, ** *p* < 0.01 (versus given NT in a HG). n = 5–6 per group. Error bars indicate the SEM derived from one-way ANOVA.

To unravel the molecular mechanisms underlying a potential estrogen-miR-10a/b-5p pathway, we conducted an analysis of interactions among miR-10a/b-5p, E2, NCOR2, insulin (INS), and INS receptor (INSR) using Ingenuity Pathway Analysis. The resulting analysis reveals that E2 indirectly interacts with the key metabolic regulators, INS and INSR, through the miR-10a/b-5p-NCOR2-INSR pathway (Figure 5a). miR-10a/b-5p directly targets NCOR2 by binding a seed region at the human NCOR2 3′ UTR [20], which is also conserved in mice (Figure 5b). For target validation, we developed a luciferase-based Ncor2-10a target validation, pLenti-Luc-10a-Ncor2 vector, incorporating the murine pre-mir-10a gene into the divided cDNAs of Luc-a and Luc-b exons, and inserting the murine Ncor2 target site of miR-10a-5p (wild type or mutant) at the end of luciferase gene (Figure 5c). Mature miR-10a-5p, generated from pre-mir-10a spliced from the Luc-a and Luc-b transcripts, would effectively target the Ncor2 wild-type target site, but not the mutant target site (Figure 5c). Robust generation of mature miR-10a-5p from the target validation vector was confirmed in HEK293T cells (Figure 5d). miR-10a-5p levels were significantly increased in HEK293T cells transfected with the pLenti-Luc-10a vector compared to those transfected with the empty pLenti-Luc vector without pre-mir-10a. The luciferase-based targeting effect of miR-10a-5p on NCOR2 was then confirmed in HEK293T cells using these target validation vectors containing the Ncor2 wild-type target site (pLenti-Luc-10a-WT) or mutant target site (pLenti-Luc-10a-Mut). Luciferase was markedly reduced in HEK293T cells transfected with pLenti-Luc-10a-WT while it was significantly increased in pLenti-Luc-10a-Mut and further increased by pLenti-Luc-10a-Mut co-transfected by miR-10a-5p inhibitor (Figure 5e). Next, we explored the effects of E2 on the expression of miR-10a/b-5p, NCOR2, INS, and INSR in NIT-2 cells (mouse pancreatic β-cells). Expression of miR-10a/b-5p in NIT-2 cells notably decreased in a high glucose medium, compared to a normal glucose medium, but gradually increased after treatment with E2 in a dose-dependent manner (Figure 5f). Consistent with findings in OVX/HFHSD diabetic female mice, INSR levels, but not INS levels, were markedly reduced in the high glucose medium, which was then restored by E2 (Figure 5g,h). Furthermore, NCOR2 expression drastically increased in cells incubated in high glucose conditions, and this increase was subsequently reduced by E2 in a dose-dependent manner (Figure 5g,h). Taken together, these findings suggest that E2 positively regulates INSR under hyperglycemic conditions via the NCOR2-miR-10a/b-5p regulatory axis.

### 2.6. Female Diabetic Patients Have Dysregulated Expression of miR-10a-5p Alongside Insulin and INSR

**Figure 6:**
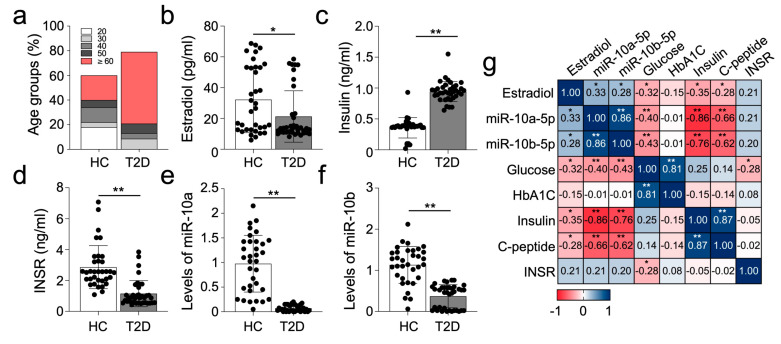
Altered levels of miR-10a/b-5p, INS, and INSR in female patients with T2D. (**a**) Distribution of age groups in female healthy control (HC) individuals and T2D patients. (**b**–**d**) Comparison of estrogen, insulin, and INSR levels in the plasma samples from female T2D patients (n = 37) compared to female HC individuals (n = 34). ** *p* < 0.01. (**e**,**f**) Levels of miR-10a-5p and miR-10b-5p in the plasma samples from female T2D patients and HC individuals. (**g**) Spearman rank correlation between levels of estradiol, miR-10a-5p, miR-10b-5p, and metabolic parameters in female T2D patients and HC individuals. Error bars indicate the SEM derived from one-way ANOVA. * *p* < 0.05, ** *p* < 0.01.

We previously showed that the miR-10a/b-5p-KLF11-KIT pathway across diabetic mice and human T2D patients is evident in pancreatic, colonic, and blood (serum or plasma) samples [16]. To further investigate this, we analyzed plasma samples obtained from T2D female patients and healthy individuals (Supplementary Table S2 for clinical characteristics), examining the expression patterns of miR-10a/b-5p, insulin, and INSR, akin to our observations in murine models. Notably, the diabetic female patient group comprised a higher proportion of postmenopausal individuals (50 and ≥60 years old) compared to the healthy control group, with lower estrogen levels observed in diabetic patients (Figure 6a,b). Corresponding to our murine findings, diabetic patient samples exhibited significantly elevated levels of glucose (161.6 mg/dL vs. 89.4 mg/dL), A1C (8.0% vs. 5.4%), C-peptide (2.1 ng/mL vs. 1.7 ng/mL), and insulin (2.35 ng/mL vs. 0.89 ng/mL) (Figure 6c, Supplementary Table S2), alongside noticeable reductions in INSR and miR-10a/b-5p expression (Figure 6d–f). Correlation analysis revealed a positive correlation between miR-10a/b-5p levels and INSR expression, while negative correlations were observed with glucose, insulin, and C-peptide in diabetic female patient samples. Additionally, estradiol levels exhibited positive correlations with miR-10a/b-5p levels, but negative correlations with glucose levels and INS expression (Figure 6g). Collectively, the levels of miR-10a/b-5p levels and the profile of key diabetic regulators in female diabetic patient samples closely resembled those found within OVX/HFHSD diabetic mice. These findings suggest that female T2D in both mice and humans is defined by INSR deficient (insulin-resistant) diabetes, regulated through an estrogen-dependent miR-10a/b-5p pathway.

## 3. Discussion

In our prior study, we highlighted the significance of the miR-10a/b-5p-mediated KIT pathway as a crucial regulator in T2D [16]. However, biological sex can differentially impact the pathogenesis of T2D. Male mice lacking these miRNAs develop T2D, but similarly lacking female mice have a delayed onset of diabetes. In this study, we unveil that miR-10a/b-5p can regulate insulin-resistant T2D through an estrogen-dependent pathway in female mice.

Estrogen is known to protect female mice from developing diabetes by preventing pancreatic β-cell damage [4,10]. This hormonal protection from diabetes is supported by the following observations: firstly, diabetes is more prevalent in middle-aged males than (estrogen-producing) females [21]; secondly, postmenopausal females who cease estrogen production exhibit a higher risk of diabetes compared to age-matched males [22]; and finally, glucose intolerance is more compromised in postmenopausal females compared to age-matched males [23].

In this study, we observed a notable difference in the manifestation of T2D between male and female mice under HFHSD conditions. Male mice fed HFHSD developed T2D around 3–4 months of age, whereas female mice fed HFHSD exhibited onset at a later stage, around 8–9 months. HFHSD mimics the Western diet, which is well-known for inducing insulin resistance and replicating the metabolic conditions associated with T2D. Additionally, our observation indicates that this diet triggers the early onset of diabetic phenotypes in estrogen-deficient OVX female mice, highlighting its pathological role in metabolic dysfunction and its significance for investigating the miR-10a/b-5p/NCOR2 pathway in females. Furthermore, our previous research demonstrated that male mice with a knockout of *mir-10b* in KIT^+^ cells developed T2D around 4 months, whereas female mice lacking *mir-10b* showed a significant delay in T2D onset, occurring around 6 months [16]. Remarkably, this delay in T2D onset was observed in female mice subjected to HFHSD and with *mir-10b* knockout disappeared when these mice underwent ovariectomy (OVX) and were fed with HFHSD. Notably, levels of miR-10a/b-5p were reduced in both normal diet (ND) and HFHSD-fed mice after OVX, while they increased with estrogen supplementation both in vitro and in vivo. These findings strongly indicate that the estrogen-dependent miR-10a/b pathway plays a crucial role in regulating the diabetic phenotype in females. Moreover, the estrogen-mediated protection against T2D diminishes in OVX/HFHSD-fed female mice, mimicking postmenopausal females who experience a decline in estrogen production, thus increasing the risk for T2D. In fact, the FDA has approved postmenopausal estrogen therapy, which delays the onset of T2D in females by improving β-cell insulin secretion, glucose tolerance, and insulin sensitivity [24]. However, our study revealed that estrogen exerts a temporary effect in lowering glucose in diabetic female mice whereas miR-10a/b-5p exhibits a prolonged effect, suggesting miR-10a/b-5p restoration is a potentially better therapeutic strategy.

Insulin resistance triggers compensatory hyperinsulinemia, exacerbating weight gain and worsening insulin resistance [25]. Obese and diabetic individuals often exhibit decreased INSR and INSR kinase activity [26]. Our investigation revealed elevated insulin levels in both female OVX/HFHSD mice and diabetic human subjects, concomitant with decreased INSR expression in both groups, indicating a reciprocal relationship between insulin resistance and INSR levels. Decreased INSR expression may lead to an increase in insulin production as a compensatory response to hyperglycemia [27]. Additionally, we observed that insulin levels were temporarily increased at the prediabetic stage in HFHSD male mice while glucose concentrations continued to rise to diabetic levels, suggesting increased insulin levels lead to insulin resistance in males as well. However, there is a biological sex difference in insulin levels between diabetic male and female mice: they are decreased in diabetic males but increased in diabetic females. Hyperglycemia in female mice with hyperinsulinemia might be attributed to insulin resistance possibly stemming from reduced INSR expression. Importantly, increased insulin production reduces INSR biosynthesis and accelerates INSR degradation [27,28].

**Figure 7:**
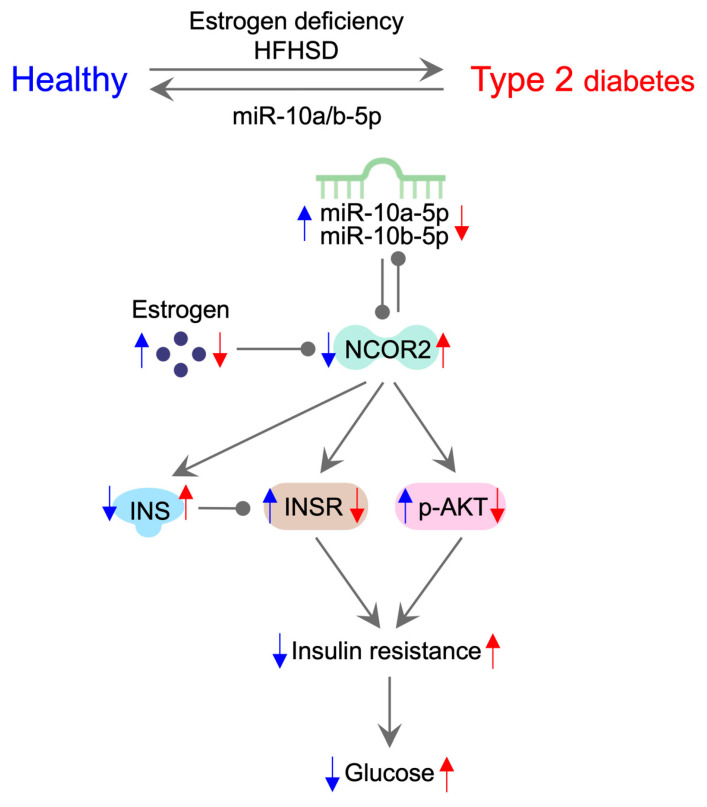
Molecular pathway outlining the etiology and pathogenesis of insulin-resistant T2D in females. Ovariectomized HFHSD-fed female mice develop T2D, which can be rescued by miR-10a-5p or miR-10b-5p injection. In healthy female mice, estrogen protects against T2D via the estrogen-dependent miR-10a/b-5p pathway. In ovariectomized HFHSD-fed female mice, estrogen deficiency reduces the expression of miR-10a/b-5p via NCOR2. The mutual inhibition between miR-10a/b-5p and NCOR2 enhances NCOR2 levels, increasing insulin production and decreasing INSR expression and AKT phosphorylation, leading to insulin resistance and T2D. Blue and red arrows denote up and down-regulation of designated miRNAs, hormones, proteins, or conditions in healthy or diabetic states.

Our study suggests that miR-10a/b-5p is pivotal in regulating T2D through estrogen-signaling pathways in females. We propose that insulin resistance and glucose homeostasis are regulated by the estrogen-dependent miR-10a/b-5p mechanism (Figure 7). In female mice, both estrogen deficiency and HFHSD exposure induce T2D by downregulating miR-10a/b-5p, while restoration of miR-10a/b-5p rescues the disease state. The decline in estrogen levels precipitates a decrease in the expression of miR-10a/b-5p through the upregulation of NCOR2. The feedback loop of reciprocal inhibition between miR-10a/b-5p and NCOR2 amplifies the further reduction in miR-10a/b-5p and the increase in NCOR2. Elevated NCOR2 levels increase insulin (INS) production, which subsequently decreases INSR expression and AKT phosphorylation (p-AKT), leading to the development of insulin resistance and ultimately hyperglycemia. This proposed model is supported by other studies: Estrogen positively regulates the expression of miR-10a/b-5p [29], which directly targets NCOR2 [20]. Estrogen functions by its receptors, ERα and ERβ, but ERα is predominantly expressed in pancreatic β cells [30,31]. ERα reduces the expression of NCOR2 (known as SMRTα) [32], while NCOR2 in turn reduces the expression of ERα [33,34,35,36,37,38,39]. These findings suggest that E2 reduces NCOR2 levels in β cells via another feedback loop of ERα.

Previous studies strongly support the reduction of NCOR2 regulatation by estrogen (Supplementary Figure S1). Estrogen negatively regulates NCOR2 levels via Sirtuin 1 (SIRT1). SIRT1 is crucial for enhancing metabolic functions against T2D [40]. Estrogen and its receptors ERα/β increase SIRT1 levels [41,42,43]. In turn, SIRT1 decreases NCOR2 expression [44]. Estrogen also enhances AMP-activated protein kinase (AMPK) [45] and peroxisome proliferator-activated receptor gamma (PPARγ) [46] through SIRT1 [47,48], both of which play a key role in T2D. AMPK further activates SIRT1 [49], creating a positive feedback loop. Additionally, SIRT1 positively regulates PPARγ [47] by modulating NCOR2, which suppresses PPARγ [50]. Our study revealed that NCOR2’s effect is amplified by the negative feedback loop of miR-10a/b-5p, highlighting the crucial role of these miRNAs in the PPARγ signaling pathway in female T2D.

The current study explored the miR-10a/b-5p-NCOR2 pathway in estrogen-deficient diabetic female mice, uncovering a gender-specific molecular mechanism of insulin resistance. Unlike previous research that predominately focused on individual miRNAs, such as miR-375 [51], miR-320a [52], and miR-103/107 [53], which are key players in insulin resistance and diabetes in male mice, our study highlights distinct molecular interactions in females. In addition, research has identified numerous dysregulated miRNAs in gestational (female) diabetes that regulate metabolic adaptations, including miR-222 [54], miR-98 [55], miR-518d [56], miR-340 [57], miR-130b and miR-148a [58], miR-33a-5p [59], miR-330-3p [60], miR-494 [61], miR-96 [62], and miR-221 [63]. Notably, miR-222 is upregulated in gestational diabetes, leading to estrogen-induced insulin resistance by targeting ERα [54]; however, this interaction has only been tested in 3T3-L1 cells [54]. Additionally, the therapeutic potential of these dysregulated miRNAs in gestational diabetes remains untested in vivo. Our study confirmed the involvement of the miR-10a/b-5p-NCOR2 pathway in both estrogen-deficient diabetic female mice and human subjects and demonstrated the efficacy of the miR-10a/b-5p mimics in this context.

The miR-10a/b-5p mimics identified in this study show potential for treating insulin resistance, especially in post-menopausal female patients with T2D. To advance from experimental models to clinical applications, several essential steps are necessary: conducting efficacy, safety, and toxicity studies in animals and non-human primates; optimizing delivery systems for human use; and evaluating efficacy and safety through clinical trials. Several miRNA-based therapeutics have advanced to clinical trials, holding significant promise for treating and preventing many human diseases [64].

There are several limitations in this study. We provide the estrogen-dependent mechanism of insulin-resistant T2D protection in females via the miR-10a/b-5p-NCOR2-INSR pathway. The mechanism was identified and tested in estrogen-present and -deficient T2D female mice and human patients. Although the prevalence of T2D in females is much higher in estrogen-deficient women (over age 45 years) compared to estrogen-present women, T2D also occurs in estrogen-present women. More studies are needed to identify estrogen-independent mechanisms or defects in the miR-10a/b-5p-NCOR2-INSR pathway in estrogen-present female patients with T2D. We examined the miR-10a/b-5p-INS-INSR axis in plasma samples from female patients with T2D and healthy donor groups. We confirmed the dysregulation of the miR-10a/b-5p-INS-INSR pathway in estrogen-deficient T2D female mice. However, further studies are warranted to verify the miR-10a/b-5p-INS-INSR pathway in other tissues including the pancreas in estrogen-deficient female patients with T2D.

In summary, our current study unveils an intricate gender- and sex-dependent mechanism governed by miR-10a/b-5p in the onset of T2D in females. Notably, our findings underscore that in female T2D cases, characterized by insulin resistance, the pathogenesis is mainly driven through the miR-10a/b-5p-NCOR2-INSR axis.

## 4. Methods and Materials

### 4.1. Animal, Diet, and Ovariectomy (OVX) Surgery

C57BL/6J male or female mice (The Jackson Laboratory, Bar Harbor, ME, USA) were fed with a normal diet (ND, Envigo, Indianapolis, IN, USA) or a high-fat, high-sucrose diet (HFHSD, Envigo, Indianapolis, IN, USA). Ovariectomies and sham surgeries were performed on four- to six-week-old female mice [16]. All animal usage and procedures were approved by the Institutional Animal Care and Use Committee at the University of Nevada-Reno (UNR) Animal Resource.

### 4.2. Body Mass and Blood Glucose Measurements

Body mass and fasting blood glucose levels were measured weekly, or bi-weekly in mice fasted for 6 h. Blood samples were collected from the tail vein, and glucose levels were measured using a blood glucose monitoring system (ReliOnTM Prime, Bentonville, AR, USA) [16].

Glucose tolerance tests (GTT) and insulin tolerance tests (ITT) were conducted to assess T2D progression [16]. For GTT and ITT, dextrose (2 g/kg body mass, Sigma-Aldrich, St. Louis, MO, USA) or insulin glargine (0.75 IU/kg body mass, Sanofi-Aventis, Paris, France) was intraperitoneally injected into mice fasted for 6 h. Glucose concentration was measured in blood collected before glucose or insulin injection (0 min) and at 30-, 60-, 90-, and 120-min post-injection.

### 4.3. Patient Samples

Human plasma samples were obtained from 37 female patients diagnosed with T2D, and 32 female healthy volunteers at the Wonkwang University Medical Center (South Korea) (Supplementary Table S2). The research participants included in this study were adults aged 21–80 years. Cases were defined as diabetic for patients diagnosed by an endocrinologist at the Wonkwang University Medical Center. All human subjects provided informed consent, and all study procedures were approved by the Wonkwang University Institutional Review Board (WKIRB-201906-BR-046).

### 4.4. Enzyme-Linked Immunosorbent Assay (ELISA)

Levels of insulin, insulin receptor (INSR), and E2 were measured in murine whole blood, serum, and human plasma samples. ELISAs were conducted on both murine and human samples using the human and mouse Insulin ELISA Kit (Crystal Chem, Elk Grove Village, IL, USA), INSR ELISA Kit (Antibodies-online, Philadelphia, PA, USA), and Estradiol ELISA kit (Abcam, Cambridge, UK), following the manufacturer’s instructions.

### 4.5. Reverse Transcription Quantitative Polymerase Chain Reaction (RT-qPCR)

Total RNAs were extracted from murine blood, pancreas, human plasma samples, and cultured cells using the mirVana miRNA Isolation Kit (Thermo Fisher Scientific, Waltham, MA, USA) as previously described [65]. Subsequently, a TaqMan probe-based qPCR assay (Applied Biosystems, Waltham, MA, USA) was performed on the isolated total RNAs [16]. TaqMan Advanced MicroRNA Assay probes were commercially obtained and used, including hsa-miR-10a-5p/mmu-miR-10a-5p (Gene ID: MI0000266), hsa-miR-10b-5p/mmu-miR-10b-5p (Gene ID: MI0000267), mmu-snoRNA55 (Gene ID: AF357318) and hsa-RNU44 (Gene ID: NR_002750). A qPCR was conducted using the CFX Connect Real-Time PCR Detection System (Bio-Rad, Hercules, CA, USA) or qTOWER3 84 (Analytik Jena, Jena, Germany). Relative transcription levels were determined using the comparative cycle threshold method, with each miRNA’s transcription levels calculated as the relative fold-change over control small nucleolar RNA (snoRNA) genes [16]. Each sample was assayed in triplicate.

### 4.6. Immunohistochemical Analysis

Murine pancreas tissue was analyzed through cryostat section staining with INS antibody (Abcam, Cambridge, UK). Imaging was performed using an Olympus FV1000 confocal laser scanning microscope and the Fluoview FV10-ASW (Olympus, Tokyo, Japan) Viewer software (v3.1).

### 4.7. β-Estradiol (E2) Injection

OVX and HFHSD-fed female mice were intraperitoneally injected with 50 µg/kg of E2 (Sigma-Aldrich, St. Louis, MO, USA). E2 was dissolved in absolute ethanol and diluted in sunflower oil (Sigma-Aldrich, St. Louis, MO, USA) to a final concentration of 50 µg/kg. Each mouse received a 100 μL intraperitoneal injection of the prepared solution.

### 4.8. In Vivo Delivery of miRNA Mimic

In vivo-*jetPEI* (Polyplus-transfection SA, Illkirch, France) and synthesized miR-10a-5p mimic (Thermo Fisher Scientific, Waltham, MA, USA) complexes were prepared according to the manufacturer’s protocol. OVX/HFHSD-fed female mice were intraperitoneally injected with the complexes containing 500 ng/body weight (g) of miR-10a-5p mimic [16].

### 4.9. Construction of a Luciferase-miR-10a-Ncor2 Target Validation Vector and Transfection

A luciferase (*Luc*)-miR-10a-Ncor2 target validation vector was generated by adapting the pLenti-CMV-Luc-10b-mKlf11 construct, which was derived from pLenti-CMV-Luc-Puro (Addgene, Watertown, MA, USA) as detailed [16]. First, the murine pre-mir-10a sequence (110 bp), flanked with the chimeric intronic donor and adaptor sequence (78 bp) was synthesized and inserted at the middle (619 bp from ATG) of the *Luc*-coding region in the pLenti-CMV-Luc-10b-mKlf11, as similarly in the method as outlined [65]. Next, a murine Ncor2 wild type target site for miR-10a-5p (10a-Ncor2 WT TS) and its corresponding mutant target site (10a-Ncor2 Mut TS) were synthesized and replaced by integrating each into the 3′ UTR region of the *Luc* gene in the pLenti-CMV-Luc-10b-mKlf11. These modifications resulted in the generation of three distinct pLenti-Luc-10a-Ncor2 vectors: pLenti-Luc-10a without a target site, pLenti-Luc-10a-Ncro2 WT TS, and pLenti-Luc-10a-Ncro2 Mut TS. The sequence accuracy and correct orientation of the pre-mir-10a and Ncor2 target site in the vector were confirmed by sequencing. Figure 5c provides a schematic illustration of the target validation mechanism and maps of the generated pLenti-Luc-10a-Ncor2 vectors.

The cell lines, NIT-2 (murine pancreatic β-cells, ATCC, Manassas, VA, USA) and HEK293T (human kidney epithelial cells, ATCC, Manassas, VA, USA) were maintained at 37 °C under 5% CO_2_ in normal or high glucose DMEM (Thermo Fisher Scientific, Waltham, MA, USA) supplemented with 10% heat-inactivated FBS (Thermo Fisher Scientific, Waltham, MA, USA) and 1% antibiotic-antimycotic (Thermo Fisher Scientific, Waltham, MA, USA). Cells were plated in 24 well plates 24 h before transfection and reached 80–90% confluency before treatment and/or transfection. Cells (NIT-2 and HEK293T) were cultured and transfected with pLenti-CMV-Puro-Luc, pLenti-CMV-Puro-Luc-Ncro2 WT TS, or pLenti-CMV-Puro-Luc-Ncro2 Mut TS along with miR-10a-5p mimic, miR-10a-5p inhibitor or a scrambled RNA (all, Thermo Fisher Scientific, Waltham, MA, USA), at a final concentration of 50 nM of miRNAs mimic and 2 µg of plasmids and incubated for 24–48 h after transfection. All cell transfections were performed using Lipofectamine RNAiMAX (Invitrogen, Waltham, MA, USA) or jetPRIME (Polyplus-transfection SA, Illkirch, France) in accordance with the manufacturer’s instructions. Cell lines were treated with 0–20 μg/mL of E2 and incubated for 24–48 h after treatment.

### 4.10. Luciferase Activity

HEK293T cells, transfected with the pLenti-Luc-10a-Ncro2 target validation plasmids above, were rinsed with 1× PBS (pH 7.5, with Ca^2+^ and Mg^2+^) and lysed using a cell culture lysis buffer (Promega; Madison, WI, USA). After removing the cell debris, the Luciferase Assay Reagent (Promega; Madison, WI, USA) was added to the lysates according to the manufacturer’s protocol. Luciferase activity was quantified using the GloMax^®^-Multi Detection System (Promega; Madison, WI, USA).

### 4.11. Capillary Western Immunoassay

Total proteins were extracted from the murine pancreas and cultured cell samples using an air-cooled bead homogenizer (Bullet Blender Storm, Next Advance; Troy, NY, USA) and RIPA Buffer (Thermo Fisher Scientific, Waltham, MA, USA). Protein concentrations were then quantified using a detergent-compatible Bradford assay. Subsequently, a Capillary Western Immunoassay was performed on the isolated protein samples (1–5 µg per lane) using the WES system (ProteinSimple, San Jose, CA, USA) with either a 12–230 kDa or 2–40 kDa separation module, following the manufacturer’s instructions [66]. Quantification analysis of the banding patterns was carried out using the Compass software (v4.0.0) for WES. All antibodies used in the Western blots are detailed in Supplementary Table S3.

### 4.12. Statistical Analysis

The experimental data were depicted as mean ± SEM. Data presented in the figures were collected from multiple independent experiments performed on different days using age- and/or sex-matched mice and humans. For comparisons in experiments, two-tailed unpaired t-tests, area under the curve calculations, and one-way analysis of variance (ANOVA) with appropriate corrections for multiple comparisons were employed. All statistical analyses were performed using GraphPad Prism (v8.0) (GraphPad Software, La Jolla, CA, USA). Statistical significance was defined as *p*-values less than 0.05 for all tests.

## Data Availability

The data supporting this study’s findings are available on request to the corresponding author.

## Supplementary Materials

The following supporting information can be downloaded at: https://www.mdpi.com/article/10.3390/ijms251810147/s1. Ref. [67] is cited in the Supplementary Materials.

## Abbreviations

M, male; F, female; T2D, type 2 diabetes; HC, healthy control; HFHSD, high-fat, high-sucrose diet; ND, normal diet; E2, β-estradiol; OVX, ovariectomized; miRNA, microRNA; 10a, miR-10a-5p mimic; 10b, miR-10b-5p mimic; NI, no injection; PI, post-injection; GTT, glucose tolerance test; ITT, insulin tolerance test.
