# Supplementary material for: miR-10a/b-5p-NCOR2 Regulates Insulin-Resistant Diabetes in Female Mice
Source: Int J Mol Sci. 2024 Sep 21;25(18):10147. doi: 10.3390/ijms251810147 (PMC11432729; doi:10.3390/ijms251810147)
Supplement: Supplementary file 1 [file ijms-25-10147-s001.zip › ijms-3198247-supplementary.pdf]

Ha *et al.* miR-10a/b-5p-NCOR2 Regulates Insulin-Resistant Diabetes in Female Mice

The supporting information contains the following items:

Supplementary Figure S1
Supplementary Tables S1–S3
Supplementary Reference

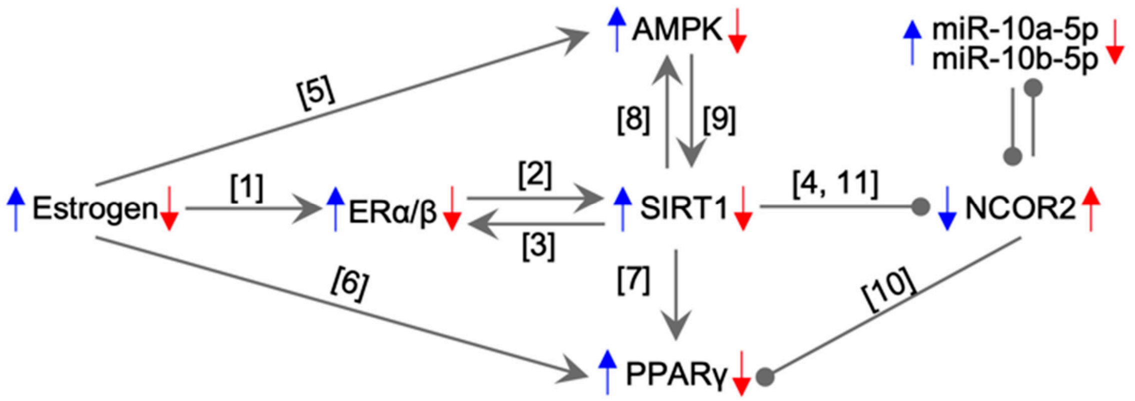

Supplementary Figure S1. Proposed molecular pathway illustrating how estrogen deficiency leads to increased NCOR2 expression, contributing to insulin resistance and type 2 diabetes (T2D) in females. This pathway is based on referenced studies supporting our findings regarding the estrogen-NCOR2-miR-10a/b-5p axis and its role in regulating insulin resistance in females. In the diagram, blue arrows represent upregulation or downregulation of hormones, proteins, and miRNAs in healthy states, while red arrows indicate in diabetic states.

Supplementary Table S1. Body weight and food intake in HFHSD mice after the injections of E2 or miR-10a/b mimic (mean ± SD)

(A) Body weight

Group		Diet	Body weight (g)							Change of body weight (g) (Before vs. PI 6W)	*P* value*
			Before	PI 1W	PI 2W	PI 3W	PI 4W	PI 5W	PI 6W		
No injection	OVX	ND	30.1±0.8	30.7±0.9	31.4±1.5	32.4±2.2	31.9±1.3	31.5±0.4	32.6±1.4	2.5±0.8	0.0542
Scrambled RNA	OVX	ND	31.9±1.5	30.1±2.2	31.5±1.3	32.4±1.4	32.2±1.1	32.6±0.5	32.9±0.2	1.0±1.1	0.1011
E2	OVX	ND	31.5±1.6	32.9±2.8	33.1±3.1	33.6±4.4	33.9±3.6	33.8±4.1	34.2±1.4	2.7±0.6	0.0863
miR-10a-5p mimic	OVX	ND	31.7±2.1	31.1±2.1	32.6±1.5	32.8±2.3	33.2±5.1	33.5±2.6	33.9±3.0	2.2±1.3	0.2476
miR-10b-5p mimic	OVX	ND	30.7±2.1	31.3±2.7	32.3±1.4	32.8±1.9	33.6±1.0	34.2±0.9	34.3±2.1	3.6±1.4	0.1210
No injection	OVX	HFHSD	48.7±3.8	49.4±3.3	49.7±5.1	50.2±5.1	50.6±5.4	51.1±2.4	52.6±3.6	3.9±2.2	0.0812
Scrambled RNA	OVX	HFHSD	49.6±2.6	49.8±2.7	50.6±2.5	51.2±2.0	51.8±1.9	52.3±1.3	52.5±1.4	2.9±1.8	0.1090
E2	OVX	HFHSD	47.2±3.6	46.9±2.3	47.7±2.7	48.1±3.6	48.6±2.1	49.0±1.0	49.9±1.3	2.7±0.9	0.0762
miR-10a-5p mimic	OVX	HFHSD	48.8±4.9	49.0±4.7	49.7±3.4	50.1±3.9	50.4±3.7	50.7±2.3	51.4±2.2	2.6±1.0	0.0563
miR-10b-5p mimic	OVX	HFHSD	47.1±2.4	48.1±1.8	48.4±3.9	49.8±3.9	49.2±2.5	50.0±2.1	50.2±1.5	3.1±1.3	0.0618

n=5-7 per group

*$p < 0.05$, **$p < 0.01$, versus before injection or given no injection in OVX HFHSD fed-female mice.

[#]PI, Post injection; W, week; OVX, ovariectomized; ND, a normal diet; HFHSD, a high-fat, high-sucrose diet; E2, 17β-estradiol

(B) Food intake

Group		Diet	Food intake (g/day)			*P* value*
			Before	PI 1W	PI 3W	
No injection	OVX	ND	4.1±1.3	3.6±0.4	3.7±0.7	0.5011
Scrambled RNA	OVX	ND	3.5±0.3	4.0±0.5	3.8±0.3	0.1002
E2	OVX	ND	3.8±0.4	3.9±0.6	4.2±0.2	0.0514
miR-10a-5p mimic	OVX	ND	3.5±0.6	3.9±0.4	3.3±0.6	0.0632
miR-10b-5p mimic	OVX	ND	4.3±0.5	4.0±0.4	3.6±0.4	0.0507
No injection	OVX	HFHSD	5.6±8.7	6.0±0.2	5.8±0.3	0.2544
Scrambled RNA	OVX	HFHSD	6.1±0.1	5.6±0.3	5.8±0.2	0.0653
E2	OVX	HFHSD	6.0±0.8	5.8±0.5	6.0±1.3	0.5874
miR-10a-5p mimic	OVX	HFHSD	4.7±1.5	4.8±0.6	5.1±0.1	0.2052
miR-10b-5p mimic	OVX	HFHSD	5.1±0.7	4.9±0.3	5.3±0.4	0.3220

n=3 per group

*$p < 0.05$, **$p < 0.01$, versus before injection or given no injection in OVX HFHSD fed-female mice.

Supplementary Table S2. Clinical characteristics of female patients with type 2 diabetes and healthy donors

Parameters	Diabetes	Healthy control
Number of cases, n	37	32
Age (years)	55 ± 6	43 ± 11
A1C (%)	8.0 ± 1.8	5.4 ± 0.5
Glucose (mg/dL)	161.6 ± 56.5	89.4 ± 11.0
Insulin (ng/mL)	2.35 ± 0.17	0.89 ± 0.4
C-peptide (ng/mL)	2.1 ± 0.6	1.7 ± 0.7
BMI (kg/m^2)	23.7 ± 3.2	22.2 ± 3.5

Supplementary Table S3. Primary antibodies used in this study

Name	Vendor (Item #)	Host	Clonality	MW	Concentration
p-AKT(Ser473)	ThermoFisher (44-621G)	Rabbit	Polyclonal	55	1:200 WES
Insulin	Abcam (ab181547)	Rabbit	Monoclonal	12	1:100 IHC, 1:200 WES
Insulin receptor	ThermoFisher (PA5-27334)	Rabbit	Polyclonal	156	1:150 WES
NCOR2	Invitrogen (PA1-843)	Rabbit	Polyclonal	250	1:50 WES
GAPDH	Cell signaling (S2118)	Rabbit	Polyclonal	36	1:500 WES

Supplementary Reference

1. Yoh, K.; Ikeda, K.; Horie, K.; Inoue, S. Roles of Estrogen, Estrogen Receptors, and Estrogen-Related Receptors in Skeletal Muscle: Regulation of Mitochondrial Function. *Int. J. Mol. Sci.* **2023**, *24*, 1853.
2. Elangovan, S.; Ramachandran, S.; Venkatesan, N.; Ananth, S.; Gnana-Prakasam, J.P.; Martin, P.M.; Browning, D.D.; Schoenlein, P.V.; Prasad, P.D.; Ganapathy, V.; et al. SIRT1 is essential for oncogenic signaling by estrogen/estrogen receptor alpha in breast cancer. *Cancer Res.* **2011**, *71*, 6654–6664.
3. Moore, R.L.; Dai, Y.; Faller, D.V. Sirtuin 1 (SIRT1) and steroid hormone receptor activity in cancer. *J. Endocrinol.* **2012**, *213*, 37–48.
4. Chen, C.; Zhou, M.; Ge, Y.; Wang, X. SIRT1 and aging related signaling pathways. *Mech. Ageing Dev.* **2020**, *187*, 111215.
5. Yang, S.; Wang, J. Estrogen Activates AMP-Activated Protein Kinase in Human Endothelial Cells via ERbeta/Ca(2+)/Calmodulin-Dependent Protein Kinase Kinase beta Pathway. *Cell Biochem. Biophys.* **2015**, *72*, 701–707.
6. Tiyerili, V.; Muller, C.F.; Fung, S.; Panek, D.; Nickenig, G.; Becher, U.M. Estrogen improves vascular function via peroxisome-proliferator-activated-receptor-gamma. *J. Mol. Cell. Cardiol.* **2012**, *53*, 268–276.
7. Li, X. SIRT1 and energy metabolism. *Acta Biochim. Biophys. Sin.* **2013**, *45*, 51–60.
8. Ruderman, N.B.; Xu, X.J.; Nelson, L.; Cacicedo, J.M.; Saha, A.K.; Lan, F.; Ido, Y. AMPK and SIRT1: A long-standing partnership? *Am. J. Physiol. Endocrinol. Metab.* **2010**, *298*, E751–E760.
9. Canto, C.; Jiang, L.Q.; Deshmukh, A.S.; Mataki, C.; Coste, A.; Lagouge, M.; Zierath, J.R.; Auwerx, J. Interdependence of AMPK and SIRT1 for metabolic adaptation to fasting and exercise in skeletal muscle. *Cell Metab.* **2010**, *11*, 213–219.
10. Yu, C.; Markan, K.; Temple, K.A.; Deplewski, D.; Brady, M.J.; Cohen, R.N. The nuclear receptor corepressors NCoR and SMRT decrease peroxisome proliferator-activated receptor gamma transcriptional activity and repress 3T3-L1 adipogenesis. *J. Biol. Chem.* **2005**, *280*, 13600–13605.
11. Kitada, M.; Koya, D. SIRT1 in Type 2 Diabetes: Mechanisms and Therapeutic Potential. *Diabetes Metab. J.* **2013**, *37*, 315–325.
